# A novel system for *in situ* determination of heat tolerance of plants: first results on alpine dwarf shrubs

**DOI:** 10.1186/1746-4811-9-7

**Published:** 2013-03-14

**Authors:** Othmar Buchner, Matthias Karadar, Ines Bauer, Gilbert Neuner

**Affiliations:** 1 University of Innsbruck, Institute of Botany, Sternwartestrasse 15, Innsbruck, 6020, Austria

**Keywords:** Chlorophyll fluorescence, Electrolyte leakage, Heat stress, *Rhododendron*, *Loiseleuria*, *Vaccinium*, Viability assessment

## Abstract

**Background:**

Heat stress and heat damage to plants gain globally increasing importance for crop production and plant survival in endangered habitats. Therefore the knowledge of heat tolerance of plants is of great interest. As many heat tolerance measurement procedures require detachment of plants and protocols expose samples to various heat temperatures in darkness, the ecological relevance of such results may be doubted. To overcome these constraints we designed a novel field compatible Heat Tolerance Testing System (HTTS) that opens the opportunity to induce controlled heat stress on plants *in situ* under full natural solar irradiation. Subsequently, heat tolerance can be evaluated by a variety of standard viability assays like the electrolyte leakage test, chlorophyll fluorescence measurements and visual assessment methods. Furthermore, recuperation can be studied under natural environmental conditions which is impossible when detached plant material is used. First results obtained on three alpine dwarf - shrubs are presented.

**Results:**

When heat tolerance of *Vaccinium gaultherioides* Bigelow was tested with the HTTS *in situ*, the visual assessment of leaves showed 50% heat injury (LT_50_) at 48.3°C, while on detached leaves where heat exposure took place in small heat chambers this already happened at 45.8°C. Natural solar irradiation being applied during heat exposure in the HTTS had significantly protective effects: In *Loiseleuria procumbens* L. (Desv.), if heat exposure (*in situ*) took place in darkness, leaf heat tolerance was 50.6°C. In contrast, when heat exposure was conducted under full natural solar irradiation heat tolerance was increased to 53.1°C. In *Rhododendron ferrugineum* L. heat tolerance of leaves was 42.5°C if the exposure took place *ex situ* and in darkness, while it was significantly increased to 45.8°C when this happened *in situ* under natural solar irradiation.

**Conclusions:**

The results obtained with the HTTS tested in the field indicate a mitigating effect of natural solar irradiation during heat exposure. Commonly used laboratory based measurement procedures expose samples in darkness and seem to underestimate leaf heat tolerance. Avoidance of detachment by the use of the HTTS allows studying heat tolerance and recuperation processes in the presence of interacting external abiotic, biotic and genetic factors under field conditions. The investigation of combined effects of heat exposure under full solar irradiation, of recuperation and repair processes but also of possible damage amplification into the results with the HTTS appears to be particularly useful as it allows determining heat tolerance of plants with a considerably high ecological significance.

## Background

High temperature is an important abiotic stress factor for plants that is not restricted to tropical areas and desert belts but also may play an important role in colder regions and at high altitudes [1]. The main reason for heat as an important stress factor in alpine sites are growth forms like rosettes and cushions that plants may have evolved to favor a decoupling from ambient cold air temperature [2]. This improvement of thermal conditions may in turn cause critical overheating as the heat trapping nature can get fatal in situations with high solar irradiation input, combined to restricted transpiration and calm winds causing situations where the thermal high temperature thresholds can get exceeded.

As high altitudes are not excepted from climate change [3] and high mountain vegetation is particularly vulnerable to it [4] especially prostrate species and plants over bare and quickly desiccating soils like many pioneer plants and their seedlings may be affected by heat stress [5-7]. This particularly, if the projected increase in global mean temperature [8] in fact should come true.

Besides natural vegetation the same applies to crop plants. Food production especially in hot and arid regions due to increased temperature has already become an agricultural problem in many areas of the world [9].

Such forecasts emphasize the urgency and the importance of a comprehensive knowledge about heat tolerance and heat stress responses [10] of plants and their capacity to adapt to an increasingly warmer climate. However, this presupposes the availability of practicable and ecologically relevant methods for the assessment of heat tolerance of plants. As is known, under natural environmental conditions heat tolerance of plants may change significantly within short time spans [11-13]. Therefore, in most cases transportation of plants from a distant natural growing site to a laboratory for conducting the heat tolerance test is not meaningful. Many heat tolerance data have been collected during the last centuries using laboratory based test procedures e.g. [14-19], however, ecologically significant data on heat tolerance of plants obtained in *in situ* measurements on plants at their specific growing site are widely missing and mechanisms of recuperation and repair appear severely understudied.

The classical method for the determination of heat tolerance is a laboratory based test procedure [20,21]. This procedure normally consists of two consecutive phases: (1) the exposure of the samples to controlled heat stress in darkness and (2) the viability assessment based on visual damage or thermal stress dependent cellular functions like cell membrane stability (electrolyte leakage) or chlorophyll fluorescence parameters (non - invasive) [22,23]. Until now only two field - portable instrument systems for the determination of heat tolerance on detached plant parts have been described [24,25]. Although with these instruments long transportation times and possible short - term alterations of heat tolerance can be avoided, they still do not allow studying after - effects and recuperation, as the samples have to be detached similar to laboratory tests. Another disadvantage of the existing field - portable systems is that heat exposure has to be conducted in darkness or under weak irradiation. This does not take into account that particularly at high altitudes midday heat usually coincides with high irradiation levels. This, however, may have significant effects on photosynthetic tissues and their ability to withstand heat stress, an aspect that has been totally neglected in the majority of earlier studies. Until today only a few studies on the determination of heat tolerance have been done, where heat stress was applied in the presence of natural solar irradiation using light transmissible tents or plastic bags e.g. [26,27] with the restriction that either temperature control was very limited or movement of plants into growth chambers [28] was necessary.

To overcome these disadvantages we designed and constructed a field compatible Heat Tolerance Testing System (HTTS) which allows *in situ* determinations of heat tolerance without the necessity to detach plant parts and with the possibility of conducting controlled heat treatments in the presence of natural solar irradiation (light - mode) and - for comparative studies - also in darkness (dark - mode). Heat treatments can be conducted under water vapor saturated air conditions that eliminate unpredictable transpirational cooling effects and ensure best possible isothermics between air and plant material. It is known [29] that heat exposure within an unsaturated atmosphere can have marked effects on the viability, because if transpirational cooling can take place, leaf temperatures will not reach the desired exposure temperature and therefore heat tolerance obtained under such conditions will possibly deviate substantially from those that are determined at 100% relative humidity.

The inclusion of recuperation and repair studies in the presence of interacting external abiotic, biotic and genetic factors under natural environmental conditions subsequent to controlled heat stress exposure powerfully enhances the ecological significance of the heat tolerance data determined with the HTTS, as they represent the *de facto* heat tolerance of a certain species at a certain developmental stage at the natural growing site. This will increase the accuracy of risk assessments related to future survival of spontaneous vegetation and will allow optimizing cultivar testing in breeding programs.

We hypothesize (1) that the presence of natural solar irradiation during short – term heat stress events may principally affect heat tolerance of leaves and (2) that heat tolerance determined on excised leaves (*ex situ*) may significantly differ from results derived from attached leaves at the natural growing site (*in situ*). Therefore, the aim of this study was to test whether heat stress in darkness or heat stress under full solar irradiation is survived better, and to observe possible influence of detachment on the measured heat tolerance. Additionally, the practical feasibility and the main features of the HTTS (*in situ* heat exposure at natural solar irradiation within a water saturated atmosphere) should be demonstrated under alpine field conditions on the basis of plant species being especially suitable for this purpose. In the following, preliminary results on selected alpine dwarf shrubs are shown obtained with the HTTS in comparison to those achieved with classical tests.

## Results

### Effects of relative air humidity on leaf temperatures

**Figure 1:**
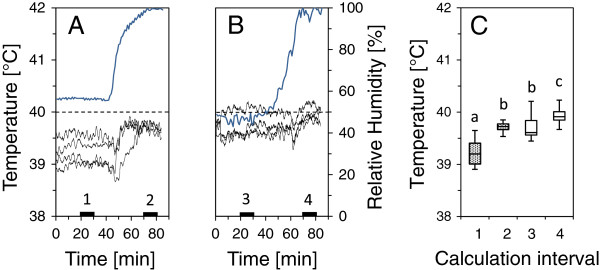
**Effects of relative humidity on leaf temperature of *R. ferrugineum *inside of two heat exposure chambers.** In the heat exposure chambers of the Heat Tolerance Testing System twigs were exposed to a sub - lethal air temperature (+40°C) that was held constant **A**. in darkness and **B**. under full solar irradiation. Thin lines: leaf temperatures, bold lines: relative humidity, dashed lines: air temperature, solid rectangles: calculation periods for statistical comparisons. At high humidity (Rh > 90%) leaf temperatures moved markedly closer to air temperature due to a strongly reduced transpiration. **C**. Box plots of the mean leaf temperature as a function of each calculation period. Boxes: median, upper and lower quartile. Whiskers: maximum, minimum. Dotted boxes: in darkness (data from A.), white boxes: at full sunlight (data from B.). At high humidity (2, 4) mean leaf temperatures had significantly (P < 0.01) increased due to the restricted leaf transpiration, while leaf temperature variability was clearly reduced.

At high but still sub - lethal temperatures and low to moderate relative humidity (Rh) inside the exposure chambers leaf transpiration has a significant cooling effect on leaf temperature and the scattering of leaf temperatures (Figure 1). This applies both to the dark - mode as well as to the light - mode. In the dark - mode and at moderate humidity (set - point temperature = 40°C, Rh = 40 - 60%) leaf temperature (mean value ± SD, n = 4) of *R. ferrugineum* was 39.2°C ± 0.2. When Rh was > 95% leaf temperature was significantly (P < 0.01) higher (39.7°C ± 0.2). At the same time the difference between leaf temperature maximum and minimum decreased from 0.7 K to 0.3 K (according to Leuzinger et al. 2010 [30] temperatures are presented in °C and temperature differences in Kelvin (K) as is the custom in bioclimatology). In the light - mode the same procedure led to a significant (P < 0.01) increase of leaf temperature from 39.7°C ±0.1to 39.9°C ± 0.1 and to a decrease between leaf temperature maximum and minimum from 0.8 K to 0.6 K. While at moderate Rh leaf temperatures were substantially lower than the set - point temperature, heat exposure of leaves at high Rh (95 - 100%) in any case led to a significant approximation of leaf temperatures to the target temperature with a simultaneous reduction of temperature scattering in terms of minima and maxima.

### Determination of heat tolerance: classical methods in comparison to the HTTS

First experiments on alpine dwarf - shrubs demonstrate that both the heat exposure mode and the viability assay may have marked effects on the measured heat tolerance values. In the following results from three representative experiments are shown:

#### Experiment (1) - *V. gaultherioides*

**Figure 2:**
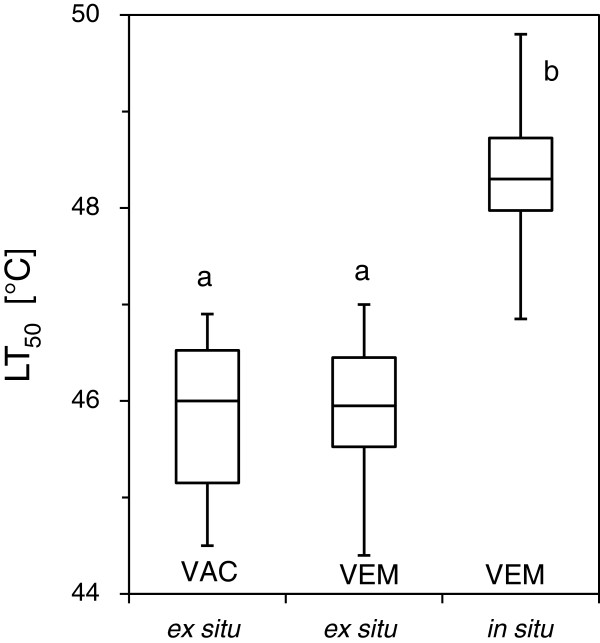
**Leaf heat tolerance of *V. gaultherioides*.** The controlled heat exposure took place on 24 July 2012 on Mt. Patscherkofel (1950 m a.s.l.). Viability assays were performed two days later. Heat tolerance (LT_50_) of detached leaves (*ex situ*) as assessed by computer based visual assessment (VAC) did not differ from results assessed by the visual estimation method (VEM), confirming that for experienced users assessment of leaf injury by visual estimation may represent a fast and viable method to assess heat damage of attached leaves. When the heat treatment was conducted *in situ* heat tolerance was significantly higher than heat tolerance that was measured on detached leaves. In both cases the controlled heat exposure took place in darkness. Boxes: median, upper and lower quartile. Whiskers: maximum, minimum. Significant differences between mean values of heat tolerance based on ANOVA and the Bonferroni - test and are indicted by different letters (P < 0.01).

To ensure comparability the exposure chambers of the HTTS were used in dark - mode and the viability was assessed by the visual estimation method (VEM) that was prior to the test calibrated and verified by comparison with a computer based digital determination of relative leaf injury (VAC) showing a high level of compliance (45.8°C ± 0.9 vs. 45.9°C ± 0.8) (Figure 2).

Leaf heat tolerance (LT_50_ ± SD) of *V. gaultherioides* when determined on detached leaves (*ex situ*) two days after a heat exposure that took place on 24 Jul 2012 in complete darkness using the exposure chambers from Buchner and Neuner (2001) [25] was 45.8°C ± 0.9. If - at the same time - the heat exposure was conducted *in situ* using the exposure chambers of the HTTS, heat tolerance was significantly (P < 0.01) higher: 48.3°C ± 0.8. This also applies to heat tolerance values carried out on the same leaves but at a later time: 48.1°C ± 1.0 (28 Jul 2012), 48.5°C ± 1.9 (2 Aug 2012).

#### Experiment (2) - *L. procumbens*

**Figure 3:**
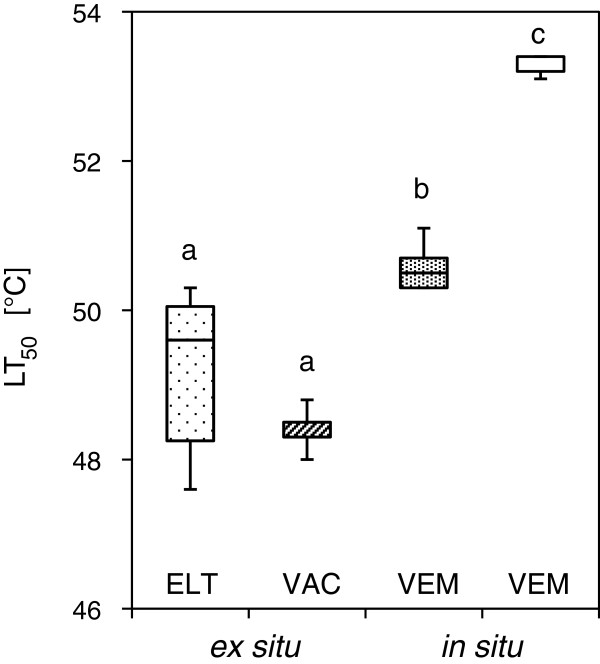
**Leaf heat tolerance of *L. procumbens.*** Heat exposure took place on 26 Sep 2011 on Mt. Patscherkofel (1960 m a.s.l.). Heat tolerance (LT_50_) determined on detached leaves (*ex situ*) using the electrolyte leakage test (ELT, dotted box) did not differ from heat tolerance as determined on detached leaves using the computer based visual assessment method (VAC, hatched box). When heat exposure was conducted *in situ* by the Heat Tolerance Testing System, heat tolerance values (fine dotted box) were significantly higher than heat tolerance values determined on detached leaves. Additionally, when the heat treatment was conducted *in situ* and under the presence of natural solar irradiation (white box) heat tolerance was significantly higher than compared to the heat tolerance as being determined from the same treatment but in darkness. For both the visual estimation method (VEM) was applied. In all cases viability assessment took place two days after the controlled heat exposure. Boxes: median, upper and lower quartile. Whiskers: maximum, minimum. Significant differences between mean values of heat tolerance based on ANOVA and the Bonferroni - test are indicated by different letters (P < 0.05).

Heat tolerance (LT_50_ ± SD) of detached leaves of *L. procumbens* (*ex situ*) as determined on 28 Sep 2011, two days after a controlled heat exposure, was 49.2°C ± 1.2 if carried out by the electrolyte leakage test (ELT), and 48.4°C ± 0.3 if determined by VAC. The difference between the results from both assessment procedures was not significant (P < 0.05). In comparison to this heat tolerance was significantly (P < 0.05) higher if determined *in situ* by the HTTS and VEM. This applies both for the heat exposure in the dark - mode (50.6°C ± 0.3) and in the light - mode (53.3°C ± 0.1). In other words: If heat exposure was conducted in darkness, LT_50_ as determined on detached leaves using ELT was significantly (P < 0.05) lower by 2.2 K than LT_50_ as determined also in darkness but *in situ*. If heat exposure was conducted *in situ* and additionally under the presence of natural solar irradiation (light - mode) this difference was even higher (4.9 K). Heat tolerance of leaves that were exposed in light - mode was higher by 2.7 K than that of leaves that had to undergo the same procedure in the HTTS in darkness (Figure 3).

#### Experiment (3) - *R. ferrugineum*

**Figure 4:**
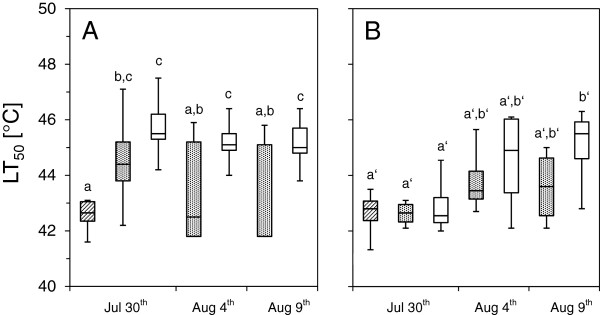
**Leaf heat tolerance of *R. ferrugineum.*** Measurements were performed on Mt. Patscherkofel (1960 m a.s.l.) using different methodical approaches with regard to the controlled heat exposure. Measurements took place on 27 July 2012 and the following assessment procedures were used: 1. Heat exposure of detached leaves (*ex situ*, in darkness), 2. *In situ* heat exposure using the Heat Tolerance Testing System (in darkness and under natural solar irradiation). **A**. Viability assessed by the visual estimation method (VEM). LT_50_ of excised leaves (hatched box) and LT_50_ determined *in situ* and in darkness (sprinkled box) did not differ significantly (exception: 30 July 2012). LT_50_ determined *in situ* under natural solar irradiation (white boxes) was always significantly higher. **B**. LT_50_ assessed by the chlorophyll fluorescence method (CHFL) showed no significant differences when detached leaves (hatched box) or attached leaves *in situ* in darkness (speckled box) were compared. LT_50_ determined *in situ* under natural solar irradiation (white boxes) was also not different except for 9 Aug 2012. Boxes: median, upper and lower quartile. Whiskers: maximum, minimum. Significant differences between mean values of heat tolerance based on ANOVA and the Duncan - test are indicated by different letters (P < 0.05).

Heat tolerance (LT_50_ ± SD) determined on leaves of *R. ferrugineum* by different heat exposure modes but identical viability assessment procedures led to significantly different results. Heat exposure took place on 27 Jul 2012. In the case of visual assessment (Figure 4A) three days after the heat exposure heat tolerance of detached leaves (*ex situ*, darkness, using the method of Buchner and Neuner 2001) [25] was 42.5°C ± 0.5 while it was significantly (P < 0.05) higher (44.5°C ± 1.4) when heat exposure was performed *in situ* and in darkness using the HTTS. If leaves were simultaneously exposed to natural solar irradiation during heat exposure (*in situ*) heat tolerance was even higher (45.8°C ± 1.0; P < 0.05). This applies also to the results obtained in viability assessments taken some days later: LT_50_ based on *in situ* heat exposure in darkness: 43.5°C ± 1.8 (4 Aug 2012) and 43.1°C ± 1.8 (9 Aug 2012) was always significantly (P < 0.05) lower than LT_50_ based on *in situ* exposure under natural solar irradiation: 45.3°C ± 1.0 (4 Aug 2012) and 45.2°C ± 0.8 (9 Aug 2012).

If viability assessment was done by chlorophyll fluorescence measurements (Fv/Fm) the differences between LT_50_ values were not as clear. However, apart from the first assessment date (30 Jul 2012) the trend was similar with the addition that in the case of dark - mode LT_50_ of detached leaves (42.6°C ± 0.8) and LT_50_ determined *in situ* (42.6°C ± 0.5; 30 Jul 2012, 43.9°C ± 1.4; 4 Aug 2012 and 43.6°C ± 1.4; 9 Aug 2012) were not significantly different (P < 0.05). Leaf heat tolerance based on heat exposure under natural solar irradiation (*in situ,* mean PPFD = 650 μMol photons · m^-2^ · s^-1^) was slightly higher (43.0°C ± 1.3; 30 Jul 2012), (44.5°C ± 1.9; 4 Aug 2012) and (45.0°C ± 1.6; 9 Aug 2012) with the restriction that only the last result (9 Aug 2012) showed a significant (P < 0.05) difference. The chlorophyll fluorescence method underestimated heat tolerance if the time span between heat exposure and viability assessment was not sufficiently long. It took eight recuperation days (4 Aug 2012) till LT_50_ values assessed by both methods had converged (Figure 4B).

## Discussion

Although a lot of literature concerning the determination of stress tolerance of plants such as heat, frost and drought is available e.g. [10,11,13,18,20-22,24,25,31-34] a complete and detailed comparison of different methods especially for determining heat tolerance of plants is still missing. As both the heat exposure mode and the method of the following viability assessment have the potential to influence the outcome, results from one and the same sample may differ significantly. We have to state that there is not only one single method that could be appropriate under all circumstances. It depends on the scientific research question and the practical feasibility which method will be preferred. For the majority of scientific questions e.g. in the fields of plant breeding, genetic engineering and crop science appropriate and well established methods may exist. This is, however, not the case for ecological issues, where heat tolerance is understood as the potential to survive heat stress on the organ - or the whole plant - level under natural conditions. Primarily, for these special purposes the HTTS has been designed. The results from the present study underline the importance of conducting heat tolerance tests *in situ* and shall increase the experimenter’s sensitivity for that issue.

### Effects of relative humidity on leaf temperature during heat exposure

When relative humidity of the air inside the exposure chambers was increased to between 95 and 100%, the difference between air and mean leaf temperature got clearly reduced. This is because of the physical laws governing leaf transpiration rate [35]. If the heat exposure chambers were operated in the dark - mode, individual leaf temperatures usually were lower than or equal to air temperature. Leaf transpiration and transpirational cooling gets quenched within a water saturated atmosphere when leaf and air temperature are the same. As transpiration rate and transpiration cooling usually is different for each individual leaf, also the differences within leaf temperatures are greater in non - saturated atmosphere than under water saturation.

### Effects of solar irradiation on leaf temperature

In light - mode transpirational cooling is superimposed by the thermal load of absorbed solar irradiation depending on irradiation intensity, individual leaf angle, leaf orientation and shading. This is why leaf temperature may exceed air temperature despite transpirational cooling. A water vapour saturated atmosphere may additionally promote overheating and therefore, convective cooling inside the exposure chamber is particularly important. Furthermore, particularly at high altitudes the impact of solar irradiation on leaf temperature can be changed immediately due to fast passing clouds. The effects of rapidly altering solar irradiation on leaf temperature places great demands on the control unit of the HTTS that needs to combine a short response time with minimal overshooting to prevent deviation of leaf temperatures from target temperature. In the case of extremely adverse environmental conditions the accuracy of the system can be improved significantly by mounting thermocouple sensors on a couple of leaves and by setting the set - point temperature to the resulting mean leaf temperature that is continuously determined by the control software.

### Effects of detachment on leaf heat tolerance

If leaves were detached (*ex situ*) this had significant effects on the determined heat tolerance value. LT_50_ of leaves from *V. gaultherioides* that had been detached before they were subjected to the controlled heat exposure was lower by -2.5 K in comparison to leaves that were exposed without detachment (see Figure 2). This applied also to *L. procumbens* where this difference was -2.2 K. Further for detached leaves the results from ELT corresponded well with the results from VAC (see Figure 3). Also for *R. ferrugineum* a significant difference in heat tolerance (-2.0 K) due to leaf detachment was detectable three days after the heat exposure (see Figure 4A).

One reason for the mainly negative effect of detachment on heat tolerance may be that recuperation and repair processes at the cellular and molecular level can run more successfully if leaves remain attached to the plant, while in the case of detachment leaves are exposed mainly to degrading processes that may amplify the pattern of heat damage. Principally, countervailing effects are also conceivable, especially if the environmental conditions subsequent to the *in situ* heat exposure and during recovery are unfavourable for recuperation (e.g. environmental stresses such as drought, frost or excessive solar irradiation). Detached leaves are commonly exposed to standard laboratory conditions with high humidity, moderate, constant temperature and irradiation intensity that largely deviate from the natural situation. Hence, the *de facto* heat tolerance of a certain plant at a certain location will be better reflected by measurements that are conducted *in situ* than under artificial laboratory conditions using detached leaves.

### Effects of irradiation mode during heat exposure on heat tolerance

Natural solar irradiation during *in situ* heat exposure increased leaf heat tolerance significantly in *L. procumbens* (+2.7 K) and in *R. ferrugineum* (+1.3 K) when compared to the results yielded after heat exposure in darkness. It could be demonstrated that solar irradiation may have a significant protective effect on heat stressed leaves. The reverse effect, a decline of heat tolerance due to the combined occurrence of heat and irradiation stress would principally also be conceivable, although our measurements do not give evidence for this.

Combined effects of irradiation and heat stress on leaf tissue are known for a long time [36-38] but the physiological mechanisms and constraints for possible protective effects of solar irradiation during heat stress are still widely unknown. As it is known that photosynthetic processes are more heat sensitive than the plasma membrane [39] in green tissue this effect possibly can be explained by photoinhibition which under certain conditions may act as a protective mechanism against heat stress [40].

However, our results demonstrate that the presence of natural solar irradiation during heat exposure is necessary for a realistic determination of heat tolerance of leaves in order to obtain results being ecologically more relevant.

Further in this experiment during the whole latent period heat tolerance based on the results of VEM (Figure 4A) differed distinctly from heat tolerance values obtained by the chlorophyll fluorescence method (CHFL) (Figure 4B). The reason for this may be that a reduction of Fv/Fm needs not to be linked exclusively to heat damage but also to a just temporary and reversible reduction of the maximum efficiency of photosystem II (PSII) - due to light, heat and other kind of stresses [23,41,42], which may considerably affect the calculation of lethal parameters such as LT_50_. Thus, CHFL, although it can be applied easily and in a non - invasive manner, cannot be recommended for determining heat tolerance without any restrictions, because it does not distinguish between photoinhibition and photodestruction. However, this shortcoming can be minimized by applying a sufficiently long recovery period between the heat exposure and the Fv/Fm measurements.

### Importance of the re - growth test

Often it may be sufficient to determine heat tolerance *ex situ* and based on detached plant parts or organs. Such methods have the advantage, that heat exposure can be conducted conveniently in the lab by avoiding natural weather conditions which often - especially at high altitudes - can be very challenging. However, if heat tolerance data with high ecological relevance are needed, heat stress must be applied under natural environmental conditions directly at the natural growing site of a plant. The advantage is that there is no need to detach the organs of interests. By this recuperation under fully natural conditions including external abiotic and biotic factors and their interactions can be studied. In frost experiments on conifers [31,32] it could be demonstrated that frost damage produced in winter took several weeks until frost damage had fully developed. If viability would have been assessed before the end of this extended latent period, frost hardiness would have been seriously overestimated. In the case of heat tolerance a similar influence of the time factor is conceivable although in summer environmental conditions, particularly temperature may be more favourable for a more rapid development of visible injuries. For example if viability is assessed by CHFL as shown for *R. ferrugineum* leaves, it can take several days (Figure 4B) or even weeks (data not shown) till PS II of heat stressed leaves is fully recovered which can have significant consequences on the calculated LT - values. In that case the assessed heat tolerance value differed by 2 K depending on the timing of the measurement after heat exposure.

With respect to the considerable amount of heat tolerance data on arctic and alpine plants that is already available e.g. [18,19] which have almost exclusively been derived from detached plant material, it can be expected that future results which will be obtained with the HTTS may significantly deviate from the existing data. Alpine vegetation is particularly prone to global climate change [4] because in alpine environments, abiotic factors, especially climate, dominate biotic interactions [43]. Changes in plant growth and reproduction [7] and upward shifts of vegetation belts are natural consequences and can already now be observed [44,45]. High mountain vegetation is at risk of drastic area losses, extinction and of disintegration of the current vegetation patterns which impacts the stability of high mountain ecosystems [4]. Especially for many pioneer plants the effects of the temperature increase may be severe [6]. Taking into account these facts, prediction models concerning alpine plant species in a global warming world will become increasingly important. Heat tolerance data determined by the HTTS may contribute to their accuracy and will sensitively affect their outcome.

## Conclusions

This work does not claim to be a comparative study of all kinds of known viability assays that are currently in use, because there is not only one ideal method that can be applied to all cases. However, being aware that viability assessment can be very difficult and resultant tolerance levels can deviate to a large extent, various methods were employed in this study. The most appropriate method concerning heat tolerance testing depends on the research question, plant species, plant organ and the level on which the heat tolerance shall be determined (cellular level, tissue level or whole plant level or with respect to certain functions such as photosynthesis, growth, fruit production or reproduction). Furthermore, the time dimension has to be considered: Is it sufficient to get a snapshot of actual heat tolerance or shall the effects of recuperation or time dependent changes of heat injuries are taken into account? Before a specific heat tolerance test assembly can be chosen, the experimenter in any case has to clarify these questions in advance before the experiments may start.

We presented a novel system for determining heat tolerance of plants *in situ* without the necessity of detachment of plant organs. It allows controlled heat exposure of plants optionally in darkness or under full natural solar irradiation. Heat exposure is conducted within a water saturated atmosphere to keep the cooling effect of leaf transpiration on individual leaf temperature minimal which ensures that the leaves have virtually the same temperature as the air in the exposure chamber. Heat tolerance exemplarily obtained for leaves of three different alpine dwarf shrubs was shown to be strongly dependent on the fact whether heat exposure was performed *in situ* or on detached leaves. Additionally, application of natural irradiation during heat exposure affected leaf heat tolerance significantly. Heat tolerance was highest when heat stress was applied *in situ* under the presence of natural solar irradiation. Incorporating damage amplification, recuperation and repair into the results, heat tolerance data determined by the HTTS are highly ecologically significant and will increase the accuracy of prediction models related to the future destiny of high alpine plants considerably.

## Methods

### Heat tolerance testing system

With the Heat Tolerance Testing System (HTTS) it is possible to determine heat tolerance of plants without the necessity of detachment. The controlled exposure to pre - defined temperatures for a unique time span can be performed *in situ*, directly at the natural growing site and under largely undisturbed natural conditions particularly with regard to solar irradiation. The system consists of a field portable supply unit, 8 exposure chambers, a netbook and external sensors for measuring leaf temperatures, relative humidity (Rh), ambient air temperature and photon flux density (PPFD). It is operated by special software providing a virtual front panel which contains all necessary control and display elements.

### Supply unit

**Figure 5:**
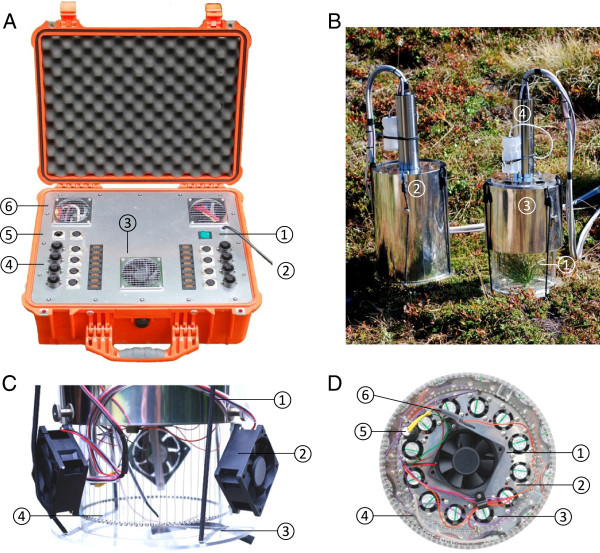
**Major components of the heat tolerance testing system. A**. The mobile supply unit is housed in a rugged and weather - proof enclosure. (1) Main switch, (2) power supply cable, (3) ventilation hole with fan, (4) connection panel for the eight exposure chambers, (5) RJ45 network port for connecting the notebook from which the complete system can be controlled, (6) venting hole (passive). **B**. Two exposure chambers in upright position during a controlled heat exposure. Heat exposure of a small *Pinus cembra* L. seedling (1) in dark - mode (2) and under natural solar irradiation (3). The handle (4) contains a hose pump and an electronic interface for controlling temperature and humidity inside the exposure chamber. **C**. Side view of an exposure chamber. (1) Sunscreen, (2) external fan for cooling purposes, (3) transparent bottom with opening slit, (4) heating wires preventing condensation inside the chamber. **D**. View into the inside of the heat exposure chamber. The main fan (1) transports air from the exposure room to the heating and humidifying system behind the separating plate (2) from where it is delivered back by 12 small fans (3). (4) Mobile temperature sensor, (5) humidity sensor (heated), (6) stationary temperature sensor.

The supply unit (Figure 5A) is built - in into a robust enclosure (PELICASE 1550, Peli Products, Provenca, Barcelona, Spain). A reconfigurable control and monitoring system (cRIO 9073: 266 MHz real time controller, NI 9264: 16 bit analog output modules, NI 9205: 16 bit analog input modules, NI 9477: 32 channel sinking digital output modules, NI 9213: 16 channel thermocouple modules; National Instruments Corporation, Austin, TX, USA) acts as a link between the software and the hardware components as relay units, sensors, fans, pumps and heating elements that shall be addressed. The processes are mainly switched and controlled either user - editable or automatically by special software based on the software platform Lab View 2010 (National Instruments Corporation, Austin, TX, USA) running on a Netbook that communicates with the system by a standard RJ 45 connector and a 10/100 Mbps Base - T Ethernet port. The software contains virtual switches, diagrams, input and output fields and virtual indicator elements. In the background of the graphical programming interface PID (proportional - integral - derivate) control algorithms assure minimal control fluctuations of the temperatures inside the exposure chambers.

### Exposure chambers

**Figure 6:**
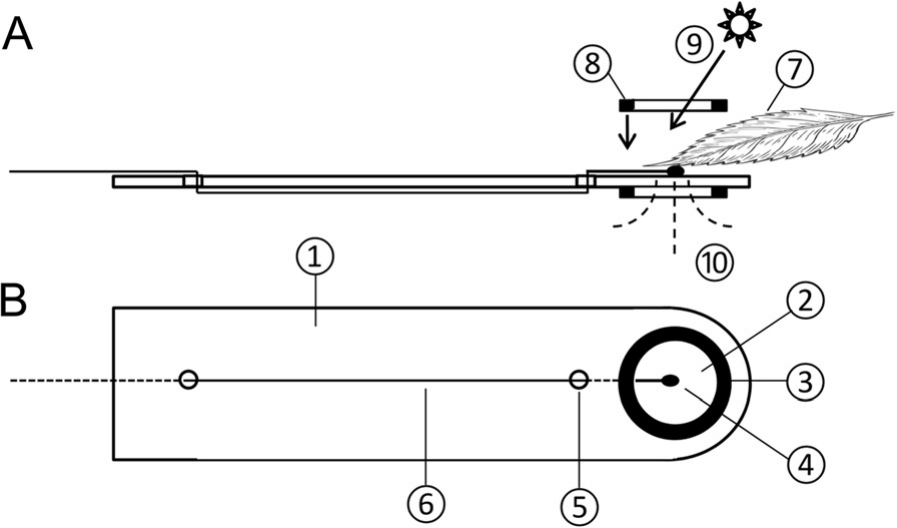
**Leaf clamp for fast attachment of a thermocouple sensor to the leaf surface. A**. side view, **B**. bottom view. The clamp consists of an elastic and thin PVC - strip (1) with an opening (2) that is surrounded by a weak, glued - on miniature ring magnet (3). Inside the opening a thermocouple sensor is placed. Two small holes in the strip (5) act as a strain relief for the thermocouple wire (6). With a second ring magnet (8) the sensor can easily be fixed to the bottom side of the leaf (7). The inner diameter of the magnets (6 mm) ensures unimpeded solar irradiation (9) and leaf transpiration (10). The leaf clamp is particularly suitable for use in wet or humid environments as heat exposure chambers and microclimate stations.

Each of the eight exposure chambers (Figure 5B-D) consists of a cylinder (150 × 250 mm) made out of Plexiglas® (2 mm, Plexiglas® XT 29070, Röhm, Darmstadt, Germany). At one end the cylinder is open to insert the plant material, while the other side is closed by a Plexiglas® cover to which a handle is mounted. This handle is hollow and contains a miniature hose pump (RP-Q1, BMT Fluid Control Systems, Frankfurt, Germany) and further electronic components as fuses, relays, semiconductors and resistors. Across its length the Plexiglas® cylinder is divided into two different areas: (1) the exposure chamber in the strict sense and (2) the heat and humidity generating compartment which contains the central heating and humidifying element. Both compartments are separated by a frame of stainless steel containing one central fan blowing air from (1) to (2) and 12 concentrically arranged fans which transport the heated and humidified air into the opposite direction. To minimize temperature gradients inside the exposure chamber these fans can be operated either continuously or in an automatically switch on - switch off mode of two crosswise arranged groups of six fans. The exposure chamber has two thermocouple sensors (Type T, solder junction diameter < 0.2 mm, TT-TI-40, Omega Engineering Inc., Stamford, US) to record the actual temperature inside the chamber; one is installed in a fixed position while the other is highly flexible and can be mounted directly on the sample surface or another area of interest (Figure 5 D). Four additional thermocouples (same size) per exposure chamber are available for being mounted on leaves or other areas of interest. These thermocouples are equipped with special leaf clamps (Figure 6) consisting of a small PVC - strip and two miniature ring magnets (R09.3x06x01.05Ni-42H, Moeller, Flensburg, Germany). With these leaf clamps thermocouple sensors can be attached permanently and in a secure manner to a variety of different leaves without injuring them and without hindering leaf transpiration. Automatic temperature control can work based on one of the two thermocouples inside each exposure chamber (Figure 5D) or on actual mean leaf temperature at an update interval of one second.

The relative humidity inside the exposure chamber is measured by a combined humidity - temperature sensor (DKRF400-10-5000, Driesen und Kern, Bad Bramstedt, Germany). The inside of the exposure chamber is covered with heating filaments (Diameter: 0.2 mm, RD 100/02, Isabellenhütte Heusler, Dillenburg, Germany) to avoid fogging that could influence spectral transmission of the Plexiglas®.

**Figure 7:**
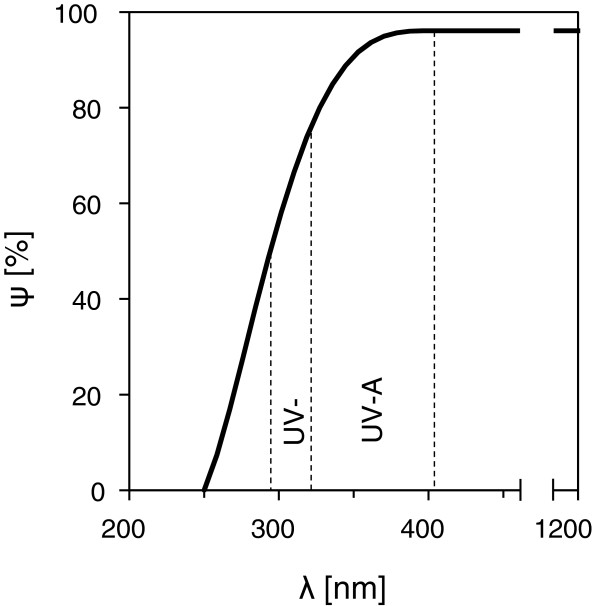
**Spectral transmittance of Plexiglas® XT Clear 20070.** The material, an extruded acrylic (polymethyl methacrylate) was used for the heat exposure chambers. For visible and near infrared light (1200 nm > λ > 380 nm) it is highly transparent (ψ = 95%). Even the physiological relevant spectral ranges of UV-A (380 nm > λ > 315 nm) and UV-B (315 nm > λ > 280 nm) are transmitted by 80% and 50%, respectively. λ wavelength, ψ spectral transmission. Source: adapted from Röhm GmbH, Darmstadt, Germany.

The exposure chamber can be closed by a circular bottom plate of Plexiglas® which is fixed by rubber - belts. For inserting the plant samples this bottom plate has a slot that can be sealed with special cellular - rubber strips. To avoid uncontrolled overheating of the exposure chambers due to solar irradiation the upper part of the Plexiglas® - cylinder is completely shielded by a sheet - plate tube out of stainless steel (thickness: 0.2 mm) that is mounted in a distance of 10 mm to the Plexiglas® - cylinder. Additionally three small fans can be mounted outside the chamber (Figure 5C). For complete darkening, special sheet - plate tubes are available that cover the whole surface. The device can thus be operated either in a “dark - mode” or in a “light - mode”. In the latter case the highly transmissible Plexiglas® ensures that all physiological relevant wavelengths from the spectrum of the solar irradiation will be transmitted to the sample without significant weakening (Figure 7).

### Plant material and experimental site

Experiments were performed on leaves of the dwarf shrubs *Vaccinium gaultherioides* Bigelow, *Rhododendron ferrugineum* L. and *Loiseleuria procumbens* L. (Desv.), growing on a north facing slope at the timberline of Mt. Patscherkofel (1960 m a.s.l., 47°12’N/11°27’E, Innsbruck, Austria) in September 2011 and July and August 2012.

### Effect of relative humidity on leaf temperature

Controlled exposure of plants to a series of defined setup temperatures, which is a prerequisite for determining heat tolerance, can be disturbed by uncontrollable leaf transpiration. A simple method to prevent transpiration is to conduct heat exposure in a vapor saturated atmosphere. For illustration of the effect of vapor saturation on leaf temperature, twigs of *R. ferrugineum* were continuously (90 min) heated inside two exposure chambers of the HTTS (chamber 1: light - mode, chamber 2: dark - mode, set - point temperature: 40°C, relative humidity: ca. 50%) while leaf temperatures were recorded. After 45 min in both chambers relative humidity was started to increase to up to 100% (45 min). Now representative periods (duration: 10 min) at unaffected and increased relative humidity were selected and leaf temperatures were analyzed and compared using statistical methods.

### Comparative determination of heat tolerance

Determinations of leaf heat tolerance were performed on *R. ferrugineum*, *V. gaultherioides* and *L. procumbens* with the objective to find out possible differences between the results carried out by commonly used methods in comparison to the HTTS. The determination of heat tolerance was always divided into two sequential steps: (1) controlled heat treatment, (2) viability assessment and calculation of lethal parameters including statistics. A summary of the different experiments including dates and details to the methods being applied is given in Table 1.

**Table 1:** Basic data concerning the comparative experiments focused on heat tolerance of alpine dwarf - shrubs

**No.**	**Species**	**Heat exposure**			**Viability assay**	
		**Date**	**Mode**		**Date**	**Method**
1	*V. gaultherioides*	24 Jul 2012	D	*ex situ*^*1*^	26 Jul 2012	VAC, VEM
		24 Jul 2012	D	*in situ*	26, 28 Jul/2 Aug 2012	VAC, VEM
2	*L. procumbens*	26 Sep 2011	D	*ex situ*^*2*^	27, 28 Sep 2011	ELT, VAC
		26 Sep 2011	D, L	*in situ*	28 Sep 2011	VEM
3	*R. ferrugineum*	27 Jul 2012	D	*ex situ*^*1*^	30 Jul 2012	VAC, CHFL
		27 Jul 2012	D,L	*in situ*	30 Jul/4, 9 Aug 2012	VEM, CHFL

Heat exposure: *ex situ* using detached leaves ^*1*^ following Buchner and Neuner (2001), ^*2*^ in water baths, *in situ,* using the Heat Tolerance Testing System HTTS. Heat exposure took place in dark - mode (D) or in light - mode (L). Viability assessment: visual assessment by computer based measurement (VAC) of leaf damage, by estimating leaf damage (VEM), by electrolyte leakage test (ELT) and by the chlorophyll fluorescence method (CHFL).

### Controlled heat treatment

For the controlled heat treatment three different methods were employed: (1) *ex situ* using temperature controlled water baths [21], (2) *ex situ* using the equipment described in Buchner and Neuner [25], (3) *in situ* using the HTTS. All these approaches have in common that leaves get exposed to a specific temperature that is held constant for a unique time span of 30 min. These specific sample temperatures were arranged in steps of 2 K difference and in a way that the lowest temperature did not cause any heat damage (0%) while the highest temperature killed the leaves (100% damage). A brief description of the three methods is given in the following:

(1) *Ex situ* - using temperature controlled water baths

Detached leaves (10 per sample) of *L. procumbens* were inserted into small plastic bags and these, in turn, were immersed into temperature controlled water baths (CC1, Huber, Offenburg, Germany) and remained there at the pre - heated target temperature for 30 min. The target temperatures were 20°C (control), 44°C, 46°C, 48°C, 50°C, 52°C, 54°C and 60°C (100% damage). To prevent transpiration of leaves during heat exposure, air was drawn out of the bags. After the heat treatment the leaves were placed on wet filter paper inside plastic bags and were exposed to room temperature and moderate irradiation conditions till the viability assessment took place.

(2) Ex situ - using the equipment following Buchner and Neuner (2001)

This method is a further development of (1) and it works basically similar to it. The difference is that detached leaves can be measured immediately after excision directly in the field avoiding transportation times to the lab. By this, alterations of actual heat tolerance during transportation can be effectively avoided. Leaves were fixed to heat stable transparencies with adhesive tape (3M™ Transpore™, 3M Österreich GmbH, Perchtoldsdorf, Austria) which were sprayed with water to prevent transpirational cooling and sandwiched between small heating plates that are housed in a transportable suit case. This method was applied to *V. gaultherioides* and *R. ferrugineum*. In both cases target temperatures were set to 20°C (control), 38°C, 40°C, 42°C, 44°C, 46°C, 48°C, 50°C, 52°C and 60°C (100% damage).

(3) *In situ* - using the Heat Tolerance Testing System HTTS

Heat exposure took place within the exposure chambers of the HTTS either in darkness (dark - mode) or under natural solar irradiation (light - mode) in a water saturated atmosphere to eliminate transpiration. Target temperatures ranged from 45°C to 51°C (*V. gaultherioides*, *R. ferrugineum*) and from 44°C to 54°C (*L. procumbens*) and were arranged in steps of 2 K to cover the whole range (0% to 100%) of damages induced by the heat treatment. The leaves could remain on the plant for the whole measurement procedure and also afterwards during the latent period before viability assessment. Viability assessment could be performed repeatedly, allowing for the inclusion of possible after effects like recuperation and stress induced acceleration of leaf senescence. With this method during the application of heat stress and in the whole latent period plants remained exposed under the natural environmental conditions.

### Viability assay

The viability of the heat treated samples was assessed by the following frequently used methods: (1) electrolyte leakage test (ELT), (2) chlorophyll fluorescence measurements (CHFL) and (3) visual assessment of leaf injury by computer based measurements (VAC) or by the visual estimation method (VEM). The common thread to all these methods was that percentage leaf injury due to a certain stress temperature to which samples had been exposed was assessed after a latent period of two to three days (or more). The resulting dose - to - effect relationship (percentage damage vs. exposure temperature) followed a classical logistic function to which the data could be easily fitted. LT_50_, the standard lethal parameter indicating the temperature that caused 50% damage, was calculated automatically (Fig. - P 2.7, Biosoft, Durham, USA). The calculation of percentage damage *X*_*T*_ [%] was based on various temperature sensitive parameters (*P*) using the following formula (1), where *P*_*T*_ is the value of *P* of a sample that had been exposed to a certain temperature *T*, *P*_*0%*_ is the value of *P* of an uninjured sample that had been exposed to a harmless temperature (T = 20°C) and *P*_*100%*_ is the value of *P* of a completely injured sample that had been exposed to a lethal temperature (T = 60°C).

(1) $$\mathrm{XT}\left[\%\right]=100\cdot \frac{P_{T}-P_{0\%}}{P_{100\%}-P_{0\%}}$$

#### (1) Electrolyte leakage test (ELT)

Principle: The ELT is a widely used and in numerous variants existing method based on the fact that heat stress affects cell membrane stability and semi - permeability causing a temperature dependent leaking of ions and solutes into the ambient solution that can be measured by an increase of electrical conductivity [20,46-49].

Experimental protocol: For *L. procumbens* subsequently to the controlled heat exposure leaves were cut in a right angle into four individual pieces and transferred into small vials (six parallels, twelve pieces per vial) which were filled with 3 ml of distilled water and 800 μg l^-1^ of dissolved gentamicin, an aminoglycoside antibiotic, to prevent microbial contamination. Subsequently these samples were infiltrated by application of a vacuum with a vacuum pump (LKC 251 E, Saskia, Ilmenau, Germany) to remove air from the intercellular spaces in the mesophyll which otherwise would have hindered the leakage of ions into the solution. After 24 h the specific electrical conductivity (G) of the leachates was determined with a conductivity meter (HD 9213, Delta Ohm, Hungen, Germany). After setting G = P, X_T%_ was calculated for each sample using formula (1).

#### (2) Chlorophyll fluorescence (CHFL)

Principle: Chlorophyll fluorescence parameters are particularly sensitive to heat stress [34,50-55]. A possible criterion for the assessment of injuries on the primary photosynthetic apparatus is F_v_/F_m_, a stress sensitive fluorescence parameter describing the maximum photochemical efficiency of Photosystem II (PS II). The stress related reduction of F_v_/F_m_ can be used as a simple tool for the assessment of leaf damage [22,33].

Experimental protocol: We determined F_v_/F_m_ on ten dark adapted leaves (30 min) before and after heat treatment (PEA MK2, Hansatech Instruments Ltd, Norfolk, UK). As a result of heat stress F_v_/F_m_ was reduced, depending on the exposure temperature. In this protocol again, X_T%_ was calculated for each sample by setting F_v_/F_m_ = − P and using formula (1).

#### (3) Visual assessment (VAC, VEM)

**Figure 8:**
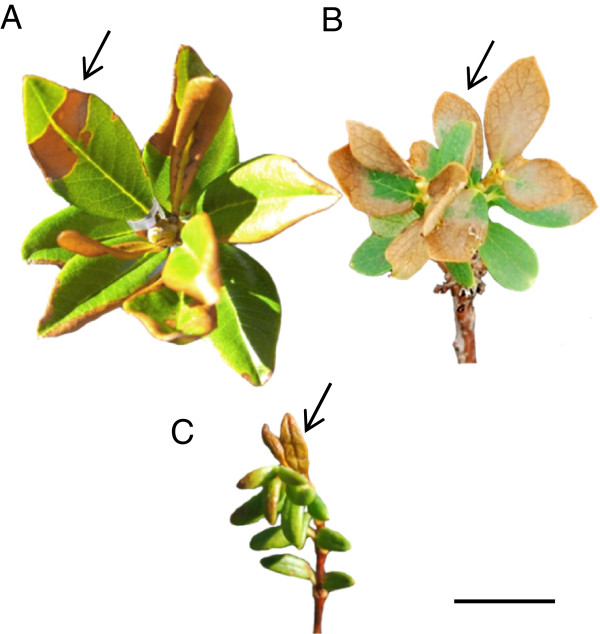
**Heat induced injuries in leaves of three alpine dwarf shrubs. A**. *R. ferrugineum*, **B**. *V. gaultherioides* and **C**. *L. procumbens*. Heat treatment was performed *in situ* with the Heat Tolerance Testing System under full natural solar irradiation during summer 2012 on Mt. Patscherkofel (1960 m a.s.l.). As in these species damaged leaf areas show typical, sharped - edged discolorations (arrows) the proportion of damaged to intact leaf area per leaf can easily be determined. Black line: 1 cm.

Principle: Due to cell membrane damage and loss of cell integrity heat stress induced injuries become apparent as discolorations on damaged leaf areas, especially if cell content is rich on phenolic compounds as e.g. in leaf tissue of many Ericaceae (Figure 8). Visual assessment methods have proved highly effective wherever leaf injuries can be detected as visible color changes [13,25,56].

Experimental protocol: For the visual assessment of heat injuries the relative proportion of damaged leaf area per leaf was determined (*V. gaulterhioides*: 32 leaves, *L. procumbens* and *R. ferrugineum*: 10 leaves). This happened by surveying leaf areas (injured areas divided to total leaf area) of scanned leaves using special graphical analyzing software (Optimas 6.5, Optimas Corp., Seattle, USA) (VAC) or by visual estimation (VEM) of percentage leaf area that was damaged. For leaves VAC normally is the method of choice. As it is not generally applicable to leaves still attached to the plant, for *in situ* measurements it was replaced by the visual estimation method (VEM) that produced similar results. The results from the visual assessment methods (VAC, VEM) were ratios between 0 and 1, which were set into formula (1) to calculate percentage damage X_T_ [%].

### Statistics

For descriptive statistical data analysis and comparisons of mean values SPSS - software (SPSS Version 18.0, SPSS Inc., Chicago, US) was used. Mean values were compared either by Student’s t - test or by analysis of variance (ANOVA) followed by post hoc tests as the Bonferroni - test or the Duncan - test.

## Competing interests

The authors declare that they have no competing interests.

## Authors’ contributions

OB initiated the project, designed and built the HTTS and the control software; OB and MK carried out the experiments and analysed the data; IB designed and constructed the magnetic leaf clamps for temperature measurement within the exposure chambers. OB wrote the manuscript, GN contributed to the manuscript and provided additional input. All authors read and approved the final manuscript.

## Funding

This work was enabled by the Austrian Science Fund (FWF - project 22158 - B16 to O. Buchner).
